# Polarization under rising inequality and economic decline

**DOI:** 10.1126/sciadv.abd4201

**Published:** 2020-12-11

**Authors:** Alexander J. Stewart, Nolan McCarty, Joanna J. Bryson

**Affiliations:** 1 Department of Biology and Biochemistry, University of Houston, Houston, TX, USA.; 2 School of Public and International Affairs, Princeton University, Princeton, NJ, USA.; 3 Centre for Digital Governance, Hertie School, Berlin, Germany.; 4 Department of Computer Science, University of Bath, Bath, UK.

## Abstract

Social and political polarization is an important source of conflict in many societies. Understanding its causes has become a priority of scholars across disciplines. We demonstrate that shifts in socialization strategies analogous to political polarization can arise as a locally beneficial response to both rising wealth inequality and economic decline. In many contexts, interaction with diverse out-groups confers benefits from innovation and exploration greater than those that arise from interacting exclusively with a homogeneous in-group. However, when the economic environment favors risk aversion, a strategy of seeking lower-risk in-group interactions can be important to maintaining individual solvency. Our model shows that under conditions of economic decline or increasing inequality, some members of the population benefit from adopting a risk-averse, in-group favoring strategy. Moreover, we show that such in-group polarization can spread rapidly to the whole population and persist even when the conditions that produced it have reversed.

## Teaser

Polarization can spread and become entrenched when inequality creates subpopulations that cannot afford risks.

## INTRODUCTION

Polarization is a social phenomenon in which a population divides into belligerent groups with rigidly opposed beliefs and identities that inhibit cooperation and undermine pursuit of a common good. Recently, “populist” movements polarized against mainstream political forces have emerged in countries as varied as the United States, United Kingdom, Brazil, Hungary, Poland, India, and the Philippines, leaving scholars, journalists, and other observers scrambling to understand the source of their support. Often, discourse has been reduced to a horse race, pitting arguments focusing on social identity, such as racial, ethnic, and nationalistic hostilities, against those concerning the economic anxieties of populist movement supporters and parties.

Adherents of both claims can find support for their arguments. Proponents of racial anxiety can offer cross-sectional and experimental evidence showing a connection between support for populist positions in the United States and United Kingdom and racial anxiety (*1*–*5*), while advocates of economic anxieties can point to negative longer-term trends in the economic and social well-being of middle class voters (*6*, *7*) and its correlation with polarized sentiments (*8*).

We suggest that these arguments should be viewed as complementary rather than as competing. Declines in economic well-being and social status that may result from inequality and economic decline may also induce changes in social behavior that trigger intragroup conflict along available cleavages. Journalistic observers have noted the complementarities between economic and racial anxiety before (*9*–*12*), while a large body of research has described the empirical relationships between legislative and affective polarization and inequality (*13*, *14*). However, formal models that describe a mechanism by which polarization can arise in response to economic hardship have been lacking.

Here, we address this deficit by developing a formal model for the dynamics of in- and out-group interactions under a changing economic environment. Adopting the framework of cultural evolution, we assume that an individual’s economic success is determined both by her interactions with others and by the underlying state of the economy. Furthermore, we assume that the behavior of successful individuals is likely to be copied by others and spread through the population. In this context, we examine the evolution of a socially acquired behavioral strategy that encodes each individual’s choice of whether to interact with those who are “like” (in-group interactions) or “unlike” (out-group interactions) the self in a variety of changing economic environments. We consider the emergence of strategies that favor in-group interactions as describing the emergence of group polarization (*15*, *16*).

In our model, in-group interactions are assumed to be less risky but offer lower rewards for success compared to out-group interactions. A range of empirical evidence supports the idea that diversity is beneficial for successful decision-making (*17*–*19*); intuitively, interactions with more diverse out-group members pool greater knowledge, applicable to a wider variety of situations. These interactions, when successful, generate better solutions and greater benefits. However, we also assume that the risk of failure is higher for out-group interactions, because of a weaker capacity to coordinate among individuals, compared to more familiar in-group interactions (*20*).

We show that under a broad range of conditions, the trade-off between risk reduction and benefit maximization decreases out-group interactions, that is, increases polarization, when a population is faced with economic decline. We show that such group polarization can be contagious, and a subpopulation facing economic hardship in an otherwise strong economy can tip the whole population into a state of polarization. Moreover, we show that a population that becomes polarized can remain trapped in that suboptimal state, even after a reversal of the conditions that generated the risk aversion and polarization in the first place.

Last, we provide support for our framework by examining the empirical relationship between inequality in the United States and levels of affective polarization: a survey measure of the mutual dislike of out-partisans, which has been posited to be related to the social group cleavages underlying the party system (*15*, *21*–*23*). Using data from the last three presidential election cycles drawn from the American National Election Study (ANES) and the Cooperative Congressional Election Study (CCES), we show that inequality and affective polarization are correlated across U.S. states, a finding consistent with recent work showing that inequality and affective polarization are correlated in a panel of developed democracies (*14*). Our work offers both a theoretical account and empirical support for the emergence of polarization as a response to economic hardship. It also suggests an explanation for the apparent difficulty in reversing polarization once it becomes established (*13*).

## RESULTS

**Fig. 1:**
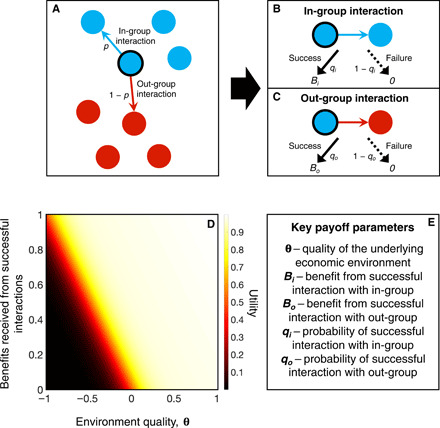
A simple model of diversity in social interactions. (**A**) From the perspective of a focal individual (black outline), a population is divided into players who are like self (blue) and unlike self (red). Each player has a strategy such that in any given interaction, they choose either a member of the in-group with probability *p* (blue arrow) or the out-group with probability 1 − *p* (red arrow). (**B**) If the focal player participates in an in-group interaction, then it is successful with probability *q_i_*, generating a benefit *B_i_* > 0. Otherwise, the interaction fails with probability 1 − *q_i_* and generates no benefit. (**C**) Similarly, if the focal player participates in an out-group interaction, then it succeeds with probability *q_o_*, generating a benefit *B*_o_ > 0. The interaction fails with probability 1 − *q_o_* and generates no benefit. (**D**) We assume that the utility or fitness for an individual from engaging in multiple social interactions follows an S-shaped curve (Eq. 1), with threshold parameter θ, which describes the quality of the underlying economic environment. When the environment quality (θ, *x* axis) and benefits received from social interactions (*y* axis) are low, utility is low (black region). When θ and/or benefits from interactions are high, utility is high (yellow region). (**E**) Five key parameters control the overall utility or fitness of an individual.

To study polarization in a population faced with rising inequality or a declining economy, we apply methods from cultural evolution and evolutionary game theory (*24*–*27*). This approach rests on the idea that each member of a large population uses a strategy *p*, which is the probability that they choose an interaction with an in-group member versus one with an out-group member. We do not make any assumptions about the nature of these interactions other than that they provide differential benefits when successful and differential probabilities of failure (Fig. 1). Similarly, we do not make assumptions about the specific identity of in- and out-groups; rather, we consider a simple base case where all subgroups in a population find in-group interactions not only less risky but also less beneficial, on average, than out-group interactions.

We define the degree of polarization in a population as the extent to which members of that population prefer in-group over out-group interactions so that maximum polarization occurs when the whole population adopts a strategy *p* = 1 and minimum polarization occurs when *p* = 0. In our analyses, we assume that populations are divided into two groups of equal size such that in-group interactions occur between randomly selected members of the same group and out-group interactions occur between randomly selected members of different groups.

In this framework, individual strategies are heritable via a copying process (*28*) in which individuals adopt the behavioral strategies of other members of the population with a probability that depends on the relative success of their respective strategies (see Methods). We assume that the copying process does not pay attention to group identity but only to the relative success of different individuals regardless of group. This assumption is conservative in that it makes polarization harder to sustain (see below).

We assume that the success of an individual’s strategy is measured by a utility function, which depends nonlinearly on the benefits received from individual interactions, as well as the state of the underlying economy (Fig. 1). We focus on a class of utility functions *w* of the following form

$$w(l_{i},l_{o})=\frac{\text{exp}[h(l_{i}B_{i}+l_{o}B_{o}+n\theta )/n]}{1+\text{exp}\;[h(l_{i}B_{i}+l_{o}B_{o}+n\theta )/n]\;}(1+\alpha (l_{i}B_{i}+l_{o}B_{o}+n\theta ))$$ (1)

where *l_i_* and *l_o_* are the number of successful in- and out-group interactions for the focal individual and *n* is the total number of such interactions attempted. *B_i_* and *B_o_* are the benefits of successful in- and out-group interactions, and θ describes the quality of the underlying economic environment so that, as the total benefits generated by a strategy approach −θ from above, the utility function becomes increasingly concave and the population becomes risk averse (Fig. 1). The parameter *h* controls the steepness of the nonlinear transition from low to high utility, and α controls the linear rate of increase of utility after the transition.

We choose this “S-shaped” utility function (Eq. 1) because it allows us to capture changes in risk aversion as the environment changes. Depending on the environment θ, the local curvature of the function can be concave, convex, or approximately linear. In addition, the S shape makes intuitive sense; the sharp decline in utility when benefits fall below −θ can be thought of as an individual dropping below a poverty line or an organization becoming insolvent. The full details and analysis of the model can be found in Methods below, and extensions to the model can be found in the Supplementary Materials (sections S2 and S3), where we consider alternative utility functions. In particular, we show that our results also hold for other commonly used concave utility functions (section S2.4).

We analyze the dynamics of polarization under two distinct sets of circumstances that have been identified previously as contributing to polarization (*13*, *14*). First, we consider a case in which the underlying economy starts to decline, lowering the standards of living for the whole population. Second, we consider the case of rising inequality in an otherwise stable economy.

In both analyses, we focus on a scenario in which the number of social interactions *n* that an individual participates in is small compared to the population or the group’s size *N*, i.e., *n* ≪ *N*. In the Supplementary Materials, we also consider the case *n* ∼ *N* and show that in a wide range of circumstances, our results continue to hold (section S2.3).

### Economic decline

Our model distinguishes the potential from the expected benefit of social interactions. The expected benefit is the probability that an interaction succeeds multiplied by the benefit it generates, i.e., *B_o_q_o_* for out-group and *B_i_q_i_* for in-group interactions. The potential benefit, by contrast, is simply the benefit received conditional on success, i.e., *B_o_* for out-group and *B_i_* for in-group interactions (Fig. 1). We assume that out-group interactions always have not only greater potential benefit, *B_o_* > *B_i_*, but also lower probability of success, *q_o_* < *q_i_*.

**Fig. 2:**
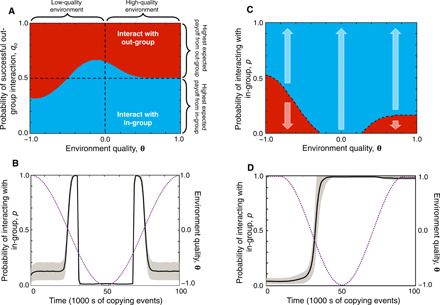
Polarization in a declining economy. Evolutionary dynamics of polarization. In the cases shown, we fix *B_i_* = 0.5, *B_o_* = 1, and *q_i_* = 1. Utility (Eq. 1) has threshold sharpness *h* = 10, and gradient α = 0.02. The number of social interactions per individual is *n* = 5. (**A**) We calculate the strategy p* that maximizes utility from Eq. 4 (see Methods). When the success of out-group interactions is independent of the strategy of the interaction partner, only one stable strategy evolves, either a highly polarized (*p* = 1, blue) or a highly diverse strategy (*p* = 0, red). (**B**) Individual-based simulations in which individuals copy more successful members of the population (selection strength σ = 10). The purple dotted line tracks the quality of the environment θ. The population size is fixed at *N* = 1000 with *q_o_* = 0.6. Shown are the mean population strategy (black line) across an ensemble of 1000 simulations and the standard deviation (SD) of the strategy distribution for the ensemble (gray region). Innovations occur at rate μ = 0.001 per copying event with size Δ = 0.01 (see section S4). (**C**) When the success of out-group interactions depends on the strategy of the interaction partner, two or more strategies can be bistable. Arrows indicate the direction of evolution in a large population, in a given environment θ. Blue regions indicate the basin of attraction for polarization p* = 1, while red regions indicate the basin of attraction for diversity p* = 0. We have set *q_o_* = 0.6. (**D**) Individual-based simulations show how polarization increases in a declining economic environment and remains even when the environment returns to being favorable. Parameters for (C) are the same as those given for (B).

Even in cases where out-group interactions have higher expected payoff than in-group ones (*B_o_q_o_* > *B_i_q_i_*), there are circumstances in which it is better to behave in a risk-averse manner and to reduce risk by choosing in-group interactions. In a prosperous, high-quality economy (θ ≈ 1), high-risk out-group interactions are favored whenever there is a greater expected payoff than that associated with in-group interactions, i.e., provided *B_o_q_o_* > *B_i_q_i_* (Fig. 2A). Thus, high-quality economies support risk taking under this model.

However, in an initially high-quality but declining environment (i.e., when θ approaches 0 from above), risk-averse strategies become increasingly beneficial, and there is a transition in the optimal behavioral strategy from out-group toward in-group interactions, i.e., toward greater polarization (Fig. 2, A and B). Intuitively, this transition occurs because, as the economy declines, failed interactions result in an increasingly sharp decline in utility, i.e., the utility function becomes increasingly concave. Thus, transitioning to low-risk interactions becomes preferable (*29*).

If the economic environment is very poor, by contrast (i.e., θ < 0), then the opposite situation can arise such that risk-tolerant behavioral strategies can become optimal (Fig. 2A, lower-left quadrant). Intuitively, this outcome occurs because, under such an environment, in-group interactions are only sufficient to produce low levels of utility, but rare successful out-group interactions can produce a sharp increase in utility, so a risk-loving “gambling for redemption” strategy becomes preferable.

The results in Fig. 2 (A and B) describe a situation in which the success of in- and out-group interactions does not depend on the strategy of the interaction partner. As a result, the population is guaranteed to evolve toward a single strategy that maximizes both individual and population payoff in that environment. However, the assumption that success of out-group interactions does not depend on the strategy of the out-group partner is unlikely to hold in general; often, the success of an interaction depends on the strategies of both participants.

In Fig. 2 (C and D), we therefore consider a case in which the success of out-group interactions depends on the strategies of both participants (see Methods). In this model, an individual *i* uses strategy *p_i_*, i.e., she seeks an in-group interaction with probability *p_i_* and an out-group interaction with probability 1 − *p_i_*. If *i* seeks an out-group interaction with another individual *j*, then we assume that the interaction succeeds with probability *q_o_*(1 − *p_j_*), where *p_j_* is the strategy of *j*. Thus, if *j* is only willing to engage in in-group interactions, i.e., *p_j_* = 1, then the out-group interaction with *i* will surely fail.

In this case, the system becomes bistable in a high-quality environment, with both a high polarization and a low polarization state possible (see section S3). However, as environmental quality declines, the low polarization equilibrium is frequently lost (Fig. 2C), and a low polarization population tends to evolve rapidly toward the high polarization state (Fig. 2D). Crucially, however, the converse does not occur for a population in a high polarization state in an improving environment. Rather, the high polarization state is always stable. The low polarization state, once lost, is thus hard to recover via a process of cultural evolution. This remains true even when the low polarization state would produce higher utility for all members of the population. Polarization, under this model, takes on the character of a social dilemma, in which there would be a collective benefit if everyone switched to a low polarization strategy, but each individual is incentivized to maintain a high polarization strategy. Recovering a low polarization equilibrium would therefore require coordination, i.e., a large portion of the population would have to simultaneously switch their strategy from in- to out-group favoring (see Discussion).

Note that our assumption that the copying process does not pay attention to group identity is conservative since it makes escape from a high polarization state easier relative to a model in which copying occurs assortatively between members of the same in-group. This is because, for a population to escape the high polarization state shown Fig. 2D, a low polarization strategy must spread to multiple members of both groups, which becomes harder if copying only occurs within an in-group.

Also note that, just as previously, a risk-tolerant gambling for redemption strategy becomes stable in a very poor environment (Fig. 2C). However, the system remains bistable, so loss of polarization is not inevitable even under these circumstances.

### Rising inequality

We have shown that economic decline can facilitate the emergence of polarization by inducing individuals to switch to more risk-averse strategies. Under rising inequality, even in an overall favorable environment, the less well-off subset of the population can face similar incentives.

We explored the effect of such inequality on the evolutionary dynamics of polarization by assuming that a fixed proportion π of the population is “wealthy” (meaning they experience a relatively good underlying economic environment, θ_+_) while the remaining 1 − π are less wealthy (i.e., they experience a relatively poor underlying economic environment θ_−_). We then parameterize the underlying economic environment experienced by the wealthy individuals as $\theta_{+}=\theta_{0}+\frac{1-\pi }{\pi }\theta_{g}$ and the environment for the nonwealthy as θ_−_ = θ_0_ − θ*_g_*, so that the average environment is πθ_+_ + (1 − π)θ_−_ = θ_0_, the Gini coefficient for the population is $g=\frac{\theta_{g}}{\theta_{0}}(1-\pi )$, and the difference in the underlying environment experienced by a wealthy versus a nonwealthy individual is θ_+_ − θ_−_ = *θ_g_*. We are now able to explore the effects of increasing inequality (increasing θ*_g_*) while keeping the average environment (θ_0_) fixed.

**Fig. 3:**
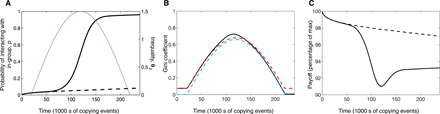
Polarization under rising inequality. We ran individual-based simulations in an economic environment with exogenously changing levels of inequality θ*_g_*. Here, we assume sinusoidally varying inequality with *a*_0_ = 1.5, θ*_g_* ∈ [0,1.1], and π = 0.1. All other parameters are those provided in Fig. 2 (C and D). (**A**) Under rising inequality θ*_g_* (purple dashed line), polarization sharply increases (black solid line) compared to the case with no inequality (black dashed line). (**B**) The Gini coefficient for the population (black solid line) changes as the inequality in the underlying economic environment θ*_g_* changes and as behavioral strategies evolve. Also shown for comparison are the Gini coefficients for a population using a fixed strategy of either polarization (*p* = 1, blue dashed line) or diversity (*p* = 0, red dashed line). (**C**) The average benefits from social interactions decline under rising inequality (solid line) and do not recover to the same levels as when inequality was absent (dashed line), even when the environment is no longer unequal.

In the case where success of out-group interactions depends on the strategy of the interaction partner, as in Fig. 2 (C and D), we find that an increase in inequality has a similar effect to a decline in the economic environment (Fig. 3A). As the environment of the less wealthy declines (Fig. 3B), they adopt risk-averse strategies, which rapidly spread to the whole population, resulting in high levels of polarization. However, the situation is not reversed when inequality declines because of the bistability of the system. Once again, we see that this irreversible polarization has the character of a social dilemma, with the average benefits gained by the population from social interactions remaining stuck in a suboptimal state (Fig. 3C). These results continue to hold in cases where the proportion of wealthy individuals is smaller (π ≪ 0.1) and where the average environment, θ_0_, and inequality, θ*_g_*, are increasing (see section S3.3).

### Affective polarization in the United States

Recent research in political science has stressed that partisanship is a salient social identity and a marker for the various other group identities that have become associated with the major political parties (*15*, *21*–*23*). A useful measure of group-based partisanship known as affective polarization is measured by the difference in “warmth” toward the preferred and nonpreferred major U.S. political parties (i.e., Republican or Democrat), reported by individuals via a feeling thermometer scale (*30*–*33*).

The effects of affective polarization are not simply attitudinal but behavioral, making this a relevant measure to consider when evaluating the predictions of our model. Previous works (*15*, *16*) have shown that partisans exhibit discriminatory behavior against opposing partisans at levels exceeding discrimination based on race. Moreover, this discrimination is manifest on many nonpolitical behaviors. For example, the authors of those studies report on the results of an experiment where respondents were asked to rate job candidates on the basis of their resume. In the experiment, the partisanship, race, and qualifications of the applicant were randomized. Partisanship played a decisive role in which job candidate was preferred. Partisan participants chose a copartisan candidate 80% of the time, but partisanship was not simple a tiebreaker. Participants chose the copartisan at high rates even when they were the less qualified candidate. In contrast, race played a much smaller role in the resume evaluations. Both white and African-American participants chose the black candidates more often than the white candidate, with African-Americans choosing the in-group candidate 18 percentage points more often than whites. In a similar vein, a recent study finds that partisan conflict (especially during preelection periods) exacerbates discriminatory behavior in taxi fare bargaining in Ghana (*34*). In summary, affective partisanship reflects a salient group identity that influences intergroup conflict and cooperation across a wide variety of economic and social domains. Therefore, given its association with identity-based politics, affective polarization is a measure well suited to evaluating the predictions of our group-based model: that inequality and intergroup conflict are correlated.

We therefore evaluate the empirical case for an association between inequality and affective polarization in the United States. Recent analysis has shown that inequality and affective polarization are correlated over time in a panel of developed democracies (*14*). Here, we use a similar approach to look at the correlation across states within the United States over the course of three presidential election cycles from 2008 to 2016, using publicly available ANES and CCES survey data (*30*–*33*).

**Fig. 4:**
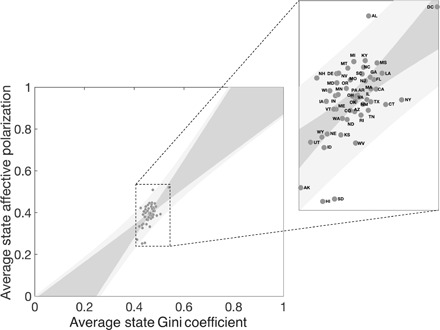
Affective polarization and inequality in the United States. We show the correlation for the pooled data across all three presidential election cycles (2008, 2012, and 2016) between state-level affective polarization estimated from (*30*–*33*) and state-level Gini coefficient taken from (*35*). The dark gray region gives the 95% confidence interval, and the light gray region gives the 95% prediction interval for the model. The expanded region shows individual state-level values. The full dataset and additional analyses can be found in section S5.

Figure 4 shows the positive correlation (significant with *p* < 0.01, two-tailed *t* test, *t* = 5.2) between state-level Gini (*35*) and the state-average affective polarization, in the pooled data across all three election cycles. A two-way fixed-effects model with election-specific intercepts gives similar results (significant positive correlation with *p* < 0.01, two-tailed *t* test, *t* = 4.4; see section S5). Additional robustness checks controlling for demographic factors, and individual-level regressions are presented in section S5 and yield similar results. Note that other factors, particularly race and education (see table S3), have equally strong or stronger effects on the degree of affective polarization in our data. Hence, future work may consider alternative measures of group cooperation and conflict in assessing the impacts of economic decline and inequality.

## DISCUSSION

Our models provide an account of polarization that connects risk aversion related to out-group interactions with declining economic well-being and increasing inequality. We now discuss limitations and possible generalizations of this account in relation to several salient questions in political economy.

First, our models may provide some insight into the emergence of populist and other far-right political movements. Central to populist ideology is a rejection of pluralism. It is based on the idea that the nation is constitutive of the dominant racial, ethnic, or religious group and that other groups seek to undermine its interests (*36*). The group polarization observed in our model can be interpreted as the emergence of populist sentiment (with the limitation that in our model, both groups are homogeneous and the same size). More generally, our work provides an account for the impact of the economic environment on the support for populist, nationalist, and other far-right movements. Consistent with our model, Majlesi *et al*. (*37*) recently showed enhanced electoral success of legislators who support a variety of group-based, right-wing causes such as immigration restriction, localized to communities negatively affected by surges in imports from China. Similarly, other authors (*38*, *39*) have shown that calamitous economic shocks such as the Great Depression and the Global Financial Crisis increased support for right-wing politicians, especially those of the far right who denigrate social out-groups. Economic stress and fear have been shown to be excellent highly localized predictors for the success of a number of populist movements including those in the Ukraine (*40*) as well as the United States and United Kingdom (*41*, *42*).

Second, in addition to connecting right-wing politics to economic decline, our model may help explain the observed causal impact of income inequality on the rise of political conservatism (*43*). While some models highlight a tendency for inequality to empower the left because of increased demands for redistribution (*44*), our work suggests a more general withdrawal from out-group interaction. Such an orientation toward the in-group over the out-group is often associated with the political right (*45*). While our model focuses on behavioral changes that lead to polarization in response to an exogenously changing environment, there are many reasons to believe that polarization, rising inequality, and economic decline can be mutually causal in reality. When people withdraw from more profitable, but riskier, out-group transactions, both aggregate and per capita output fall. This could be expected to have a self-reinforcing effect as the fall in economic output engenders even lower levels of out-group interaction. These positive feedback loops are similar to those describing interactions between ideological polarization and economic inequality (*13*). This observation provides a natural direction for future work.

Third, our model is also consistent with work highlighting the role of economic shocks in civil and social conflict (*46*, *47*). Chassang and Padro-i Miquel (*48*) have modeled the impact of economic shocks on civil war onset and suggest that shocks produce conflict because they reduce the opportunity cost of fighting in the short run. This perspective, like our model, links changes to the costs and benefits of interacting with out-groups to the state of the economic environment. Exploring how these different incentives associated with intergroup interactions interact and play out will be an important direction for future work.

Our model may also speak to debates on radicalization and political violence. While most findings indicate that individual terrorists are unlikely to be drawn from the most economically deprived sectors, poor economic conditions are often correlated with higher rates of political violence (*49*–*52*). Moreover, our model may help explain recent work showing that right-wing terrorism associated with group-based grievances is more sensitive to economic conditions and inequality than violence from left-wing groups promoting more universalistic ideologies (*53*).

Fourth, our model may contribute to ongoing debates about the origins and nature of social identity. In that our model describes the emergence of polarization in terms of a loss of intragroup interactions as a response to economic hardship, it has similarities to realistic conflict theory (RCT), which postulates that economic hardship can induce greater intergroup hostility by enhancing competition over scarce resources. RCT predicts a correlation between aggregate economic output and group-level cooperation. Similarly, under our model, out-group interactions are more likely to be favored when the economic environment is good. Thus, our model and RCT are both consistent with findings that diversity can reduce public goods investment when countries suffer economic stress (*54*). However, the assumptions underlying our model are distinct from those underlying RCT. Instead of postulating direct conflict over scarce resources, we assume that behavioral changes leading to greater polarization during economically challenging periods are driven by risk aversion. Of course, both intergroup competition for resources and increased risk aversion for out-group interactions can occur at the same time and may reinforce one another, leading to even greater entrenchment of polarized attitudes.

Last, perhaps the most important result arising from our work is that we show how easy it can be for polarization to become entrenched once it spreads through the population. Our work highlights an asymmetry in how easy it can be to move by gradual change from low to high polarization and back. The persistence of a high polarization equilibrium in our model can be viewed as the result of a coordination problem, where although most individuals would benefit if the population moved to a low polarization equilibrium, those benefits only manifest once enough individuals adopt the low polarization strategy. No one has the incentive to make the first move, a key feature of collective action problems. Since a population cannot easily escape a state of high polarization by individuals adapting their behavior, an external event may be needed to provide the necessary coordination to escape polarization. During national crises such as wars, states have very strong incentives to solve these problems. Thus, exogenous events that reestablish the importance of larger-scale (e.g., national) identities may be required to regenerate intergroup cooperation and reduce polarization. This argument is supported by a review of 20 studies (*55*), which show increased altruism in populations after experiencing war. Recent work by Scheidel *et al*. (*56*) also suggests that external shocks such as war are a necessary condition for reducing inequality. The necessity of these shocks is consistent with the findings of Scheve and Stasavage (*57*) who show that progressive taxation has largely been the product of fairness norms forged during wartime. Similarly, Jong *et al*. (*58*) show that other negative shocks such as terrorist attacks can also lead to identity fusion. Preliminary evidence also indicates that the COVID-19 pandemic, and the experience of lockdowns in particular, can increase political support for existing national leaders (*59*).

Better than morbidly waiting for further economic crises or war, we will end by pointing out an obvious recommendation as to what political leaders and governments should do to prevent persistent group polarization. Our work unambiguously highlights the importance of building and maintaining a social safety net. Social institutions may serve as a means to provide redistribution, thus reducing inequality, but our work emphasizes another important role: preventing the income of groups from falling sufficiently far to trigger the risk aversion that might lead to persistent group polarization.

## METHODS

To capture variation in behavioral strategies and its consequences for polarization, we adopt a model derived from the study of cultural evolution and evolutionary game theory where individuals accumulate benefits through multiple interactions with other members of a finite population of *N* individuals.

We assume that each individual is faced repeatedly with the choice of interacting either with someone who is like them (in-group interactions) or unlike them (out-group interactions), where in-group interactions provide a benefit *B_i_* with a probability *q_i_* and benefit 0 with probability 1 − *q_i_* (Fig. 1). That is, the risk of failure in an in-group interaction is 1 − *q_i_*. Similarly, an out-group interaction provides a benefit *B_o_* with probability *q_o_* and benefit 0 with probability 1 − *q_o_*. As discussed above, in general, we make the key assumption that out-group interactions come with higher reward (*B_o_* > *B_i_*) but also higher risk (*p_o_* < *q_o_*).

Each individual is assumed to participate in *n* interactions, whose success or failure determines the total payoff accumulated by the individual during that time period, where the number of available in- and out-group interactions is assumed very large and, consequently, *N* ≫ *n*. Typically, we assume *n* < 10, reflecting an individual who is making a decision based on a few sources of information. We discuss the case of larger numbers of in- and out-group interactions *n* in the Supplementary Materials. Each individual is then characterized by a strategy *p*, which gives the probability that they choose an in-group interaction and, consequently, each individual chooses an out-group interaction with probability 1 *− p* (Fig. 1).

Given this model, the probability that a player with strategy *p* engages in *l_i_* successful in-group interactions out of a total *k* in-group interactions and *l_o_* successful out-group interactions out of a total *n − k* out-group interactions is given by

$$\pi (k,l_{i},l_{o}|n)=(\begin{array}{c} n\\ k \end{array})p^{k}{\left(1-p\right)}^{n-k}(\begin{array}{c} k\\ l_{i} \end{array})q_{i}^{l_{i}}{\left(1-q_{i}\right)}^{k-l_{i}}(\begin{array}{c} n-k\\ l_{o} \end{array})q_{o}^{l_{o}}{\left(1-q_{o}\right)}^{n-k-l_{o}}$$ (2)

That is, the number of in- and out-group interactions and the number of successful interactions each follow binomial distributions. The resulting expected benefit derived from successful interactions under this model is then simply

$$\overset{n}{\underset{k=0}{\Sigma }}\overset{k}{\underset{l_{i}=0}{\Sigma }}\overset{n-k}{\underset{l_{o}=0}{\Sigma }}\pi (k,l_{i},l_{o}|n)(B_{i}l_{i}+B_{o}l_{o})={\mathrm{nB}}_{i}q_{i}p+{\mathrm{nB}}_{o}q_{o}(1-p)$$ (3)

and the strategy that maximizes Eq. 2 is either *p* = 1 (always interact with in-group) if *B_i_q_i_* > *B_o_q_o_* and *p* = 0 (always interact with out-group) otherwise. However, such a linear model does not, in general, reflect the reality of the way benefits accumulate in human society. In many situations, a minimum level of resources is required to achieve a particular goal (e.g., avoid starvation or reproduce in a biological system; purchase property, or start a business in an economy). Income above that threshold, while still advantageous, is less beneficial. Thus, benefits tend to accumulate nonlinearly.

At the same time, the economic environment can be influenced by exogenous factors so that the per capita resources available for each individual vary over time. When such fluctuations occur, the nonlinear accumulation of benefits described above may lead to changes in the curvature of the utility function of a given individual and, thus, their level of risk aversion. Since in- and out-group interactions differ both in their level of expected benefit and their level of risk, this leads to changes in behavior. We consider the evolutionary dynamics of behavior both in the case where the risk of out-group interactions is fixed 1 − *q_o_* and where it depends on the willingness of out-group members to engage in such interactions, i.e., where the risk associated with out-group interactions depends on the strategy adopted by other members of the population.

To understand the consequences of shifting environments and nonlinearly accumulating benefits on individual behavior in our model, we consider the evolutionary dynamics of the system. We consider a population evolving under a copying process (*28*) in which individuals are able to observe the fitness, i.e., the total benefit accumulated via in- and out-group interactions, of other individuals and compare it to their own. Note that we use the term fitness and utility interchangeably in the context of our model. The dynamics of the model are as follows: An individual *f* is chosen at random from a population of fixed size *N*. A second individual *g* is then chosen at random for her to observe. If *f* has fitness *w_f_* and *g* has fitness *w_g_*, then *h* chooses to copy the strategy of *g* with probability 1/(1 + exp [σ(*w_g_* − *w_f_*)]), where σ scales the “strength of selection” of the evolutionary process. Note that if *w_g_* ≫ *w_f_*, then the probability of *f* copying the behavior of *g* is close to 1, whereas if *w_g_* ≪ *w_f_*, then the probability is close to 0.

To explore the evolutionary dynamics of the system, we must also specify how fitness *w* depends on the benefits received from individual in- and out-group interactions, *B_i_* and *B_o_*. To model the nonlinear accumulation of fitness benefits from diverse social interactions across a range of environments, we assume that the linear accumulation of fitness benefits is modified by a sigmoidal function (Eq. 1), where *h* controls the steepness of the sigmoid (how sensitive fitness is to changes in accumulated benefits), α controls the rate of linear accumulation of benefits, and θ controls the environment, so that when θ is large (relative to accumulated benefits) and positive, the sigmoidal term is close to 1 and fitness tends to accumulate linearly. Conversely, when θ is large and negative (relative to accumulated benefits), fitness tends to be close to 0. The form of Eq. 1 reflects an environment in which a certain minimum level of benefit from social interactions (≈−θ) is required for success or survival.

From Eqs. 1 to 3, we can calculate the expected fitness $\hat{w}$ of a player with strategy *p*, under the model with fixed risk, which is simply

$$\hat{w}=\overset{n}{\underset{k=0}{\Sigma }}\overset{k}{\underset{l_{i}=0}{\Sigma }}\overset{n-k}{\underset{l_{o}=0}{\Sigma }}\pi (k,l_{i},l_{o}|n)\frac{\text{exp}\;[h(l_{i}B_{i}+l_{o}B_{o}+n\theta )/n]}{1+\text{exp}\;[h(l_{i}B_{i}+l_{o}B_{o}+n\theta )/n]}(1+\alpha (l_{i}B_{i}+l_{o}B_{o}+n\theta ))$$ (4)

To characterize the evolutionary dynamics of this system, we use Eq. 4 to determine how the strategy p* that maximizes Eq. 4 varies with the environment, θ, and the probability of success in interactions with in- and out-group members, *q_i_* and *q_o_*. Since Eq. 4 cannot be treated analytically in general, we numerically calculated the strategy p* that maximizes fitness as a function of the environment and the probability of successful in- and out-group interactions and show that for a given environment and risk level, there is a single global optimal strategy for the system [see Fig. 2 (A and B) and the Supplementary Materials].

Last, we consider a version of our model that includes the possibility that the success of out-group interactions depends on the strategy adopted by the out-group player. We assume, for simplicity, that in-group members are always willing to interact. We then assume that a successful out-group interaction between two players *g* and *f* depends on both players’ willingness to interact, i.e., on *p_g_* and *p_f_*. That is, we set $q_{o}^{\mathrm{gf}}=q_{o}(1-p_{f})$, where *q_o_* is the intrinsic probability of success and (1 − *p_f_*) is the probability that player *f* agrees to participate in the interaction. To explore the evolutionary dynamics of this system, we adopt the framework of adaptive dynamics (*60*, *61*) to calculate the stable strategies of the model under small changes to a player’s strategy *p*. The fitness of a strategy *p_f_* in a population of players using a resident strategy *p* is

w^f=Σk=0nΣli=0kΣlo=0n−k(nk)phk(1−ph)n−k×(kli)qili(1−qi)k−li(n−klo)(qo(1−p))lo(1−qo(1−p))n−k−lo×exp[h(liBi+loBo+nθ)/n]1+exp[h(liBi+loBo+nθ)/n](1+α(liBi+loBo+nθ)) (5)

and we can calculate the stability of the resident strategy *p* to invasion by calculating the selection gradient

$$s={\frac{\partial {\hat{w}}_{f}}{\partial p_{f}}|}_{p_{g}=f}$$ (6)

which determines the local evolutionary dynamics of the system. Once again, we explore the equilibria of the system numerically and show that the system is frequently bistable (Fig. 2, C and D), and for some, parameter choices have three stable equilibria (see the Supplementary Materials).

### Invasion

We consider the evolutionary dynamics under the copying process as described above (*28*), under which the probability that a player with strategy *g* copies the strategy of another player *f* is

$$r_{g,f}=\frac{1}{\left(1+\text{exp}[\sigma (w_{g}-w_{f})]\right)}$$ (7)

and the resulting growth rate of a rare mutant *f* in a population with resident strategy *g* is

$$S(f,g)=\frac{r_{g,f}}{r_{f,g}}=\frac{\left(1+\text{exp}[-\sigma (w_{g}-w_{f})]\right)}{\left(1+\text{exp}[\sigma (w_{g}-w_{f})]\right)}=\text{exp}[-\sigma (w_{g}-w_{f})]$$ (8)

Switching without loss of generality to log fitness (and ignoring the proportionality constant), we can then simply write

$$s(f,g)=w_{f}-w_{g}$$ (9)

where if *s* > 0, then *h* is increasing in frequency. To construct pairwise invasibility plots (see sections S2 and S3), we then simply look at the sign of Eqs. 8 and 9 when *w* is given by Eqs. 3 to 5. Note that in the first case, we analyze (Eqs. 3 and 4) that the payoff *w* depends only on the focal player’s strategy (i.e., the fitness of the resident and the mutant do not depend on one another). This case is formally similar to an optimal foraging model with a sigmoidal functional response curve.

A strategy *f* = *g* = g* is a local evolutionary stable strategy (ESS) if and only if

$$\frac{\partial^{2}s(f,g)}{\partial p_{f}^{2}}<0$$ (10)

when evaluated at g*, which must be a point of zero selection gradient. The strategy g* is convergence stable if and only if (*60*)

$$\frac{\partial^{2}s(f,g)}{\partial p_{g}^{2}}>\frac{\partial^{2}s(f,g)}{\partial p_{f}^{2}}$$ (11)

when evaluated at $p_{f}=p_{g}=p_{g}^{*}$. We use Eqs. 9 to 11 in constructing invasibility plots and determining the character of singular points (see sections S2 and S3).

## Supplementary Material

http://advances.sciencemag.org/cgi/content/full/6/50/eabd4201/DC1

Adobe PDF - abd4201_SM.pdf

Polarization under rising inequality and economic decline

## SUPPLEMENTARY MATERIALS

Supplementary material for this article is available at http://advances.sciencemag.org/cgi/content/full/6/50/eabd4201/DC1
