# Supplementary material for: Polarization under rising inequality and economic decline
Source: Sci Adv. 2020 Dec 11;6(50):eabd4201. doi: 10.1126/sciadv.abd4201 (PMC7732181; doi:10.1126/sciadv.abd4201)
Supplement: http://advances.sciencemag.org/cgi/content/full/6/50/eabd4201/DC1 [file supp_6_50_eabd4201__index.html]

Science Advances | Science AdvancesAAASSearchScience AdvancesMenu

## Supplementary Materials

# Polarization under rising inequality and economic decline

Alexander J. Stewart, Nolan McCarty, Joanna J. Bryson

Download Supplement

**This PDF file includes:**

- Sections S1 to S5
- Tables S1 to S3
- Figs. S1 to S11

**Files in this Data Supplement:**

- Adobe PDF - abd4201\_SM.pdf
