# Supplementary material for: Polarization under rising inequality and economic decline
Source: Sci Adv. 2020 Dec 11;6(50):eabd4201. doi: 10.1126/sciadv.abd4201 (PMC7732181; doi:10.1126/sciadv.abd4201)
Supplement: http://advances.sciencemag.org/cgi/content/full/6/50/eabd4201/DC1 [file supp_6_50_eabd4201__1.pdf]

Science Advances
AAAS

advances.sciencemag.org/cgi/content/full/6/50/eabd4201/DC1

Supplementary Materials for

Polarization under rising inequality and economic decline

Alexander J. Stewart*, Nolan McCarty*, Joanna J. Bryson*

*Corresponding author. Email: alstew@sas.upenn.edu (A.J.S.); nmccarty@princeton.edu (N.M.); jjb@alum.mit.edu (J.J.B.)

Published 11 December 2020, *Sci. Adv.* **6**, eabd4201 (2020)
DOI: 10.1126/sciadv.abd4201

This PDF file includes:

Sections S1 to S5
Tables S1 to S3
Figs. S1 to S11

In this supplement, we relax several of the modeling assumptions presented in the main text to demonstrate the robustness of our conclusions. In particular we vary the "functional response curves" that relate the outcome of a given social interaction to the accumulated utility of many interactions and the number of interactions among individuals. We also provide further details of the individual-based simulations presented in the main text. Note that equation numbers continue from the main text.

1 Model Parameters

The parameters associated with the model presented in Figure 1 and the main text are summarized in Table 1 below. We now discuss how variation in the parameters of the model impacts the associated evolutionary dynamics and the degree of polarization that arises across environments. We first discuss the case where the probability of success of an out-group interaction is constant and independent of the strategies adopted by other members of the population. We then discuss the case where the risk of out-group interactions depends on the strategy of the target for the interaction. We analyze both models by looking at the equilibria under both local and non-local mutations (i.e under scenarios where players adjust their behavior either gradually or in sudden-large shifts such as may occur in response to structural change).

Parameter	Default Simulation Value	Meaning
B_i	0.5	Benefit received due to a successful in-group interaction.
B_o	1.0	Benefit received due to a successful out-group interaction.
q_i	1.0	Probability of a successful in-group interaction
q_o	0.6	Probability of a successful out-group interaction
n	5	Number of attempted interactions before strategy update
N	1000	Population size
θ	$[-1, 1]$	Quality of the environment (no inequality)
θ_0	$[0, 1]$	Average quality of the environment (with inequality)
θ_g	$[0, 1.1]$	Strength of inequality
π	0.1	Proportion of the population that is very well off
h	2	Steepness of the functional response curve
α	0.01	Slope of the functional response curve
σ	10	Selection strength
μ	0.0001	Mutation rate

Table S1 – Model parameters and the default values chosen for main text individual-based simulations

2 Case 1: Non-social payoffs

2.1 Stability and Invasibility

We first consider the case in which the probability of success of an out-group interaction is simply q_o, which does not depend on the strategy of the target of the interaction. Thus the payoff w_f of a mutant f does not depend on the background g into witch it is introduced. This means that a strategy that maximizes w can always invade and can never be invaded, so that under an evolutionary process with non-local mutations as shown in Figure 2 (main text) the population will always arrive at the global maximum.

However, we are also interested in the behavior of the system under local mutations (or "gradual methods" as they are called in the main text). To this end we look at the selection gradient of main text Eq. 4 which gives

$$\begin{aligned}\frac{\partial s(f,g)}{\partial f} = &\sum_{k=0}^{n}\sum_{l_i=0}^{k}\sum_{l_o=0}^{n-k}\binom{n}{k}p_f^{k-1}(1-p_f)^{n-k-1}(k-np_f)\times \\ &\binom{k}{l_i}q_i^{l_i}(1-q_i)^{k-l_i}\binom{n-k}{l_o}(q_o)^{l_o}(1-q_o)^{n-k-l_o}\times \\ &\frac{\exp[h(l_iB_i+l_oB_o+n\theta)]}{1+\exp[h(l_iB_i+l_oB_o+n\theta)]}(1+r(l_iB_i+l_oB_o))\end{aligned} \tag{12}$$

which we can evaluate numerically to calculate the points of zero selection gradient as shown in Figure S1 below. We can also use Eq. 12 to gain insight into the dynamics of polarization on display in Figure 2 of the main text.

In particular, if the environment is sufficiently bad that $l_iB_i+l_oB_o+n\theta<0\ \ \forall\, l_i,l_o$ or if it sufficiently good such that $l_iB_i+l_oB_o+n\theta>0,\ \ \forall\, l_i,l_o$ then we can approximate the sigmoidal term in Eq. 12 as a constant and recover selection gradient

$$\begin{aligned}\frac{\partial s(f,g)}{\partial p_f} &= \sum_{k=0}^{n}\binom{n}{k}p_f^{k-1}(1-p_f)^{n-k-1}(k-np_f)(1+nrq_oB_o+kr(q_iB_i-q_oB_o)) \\ &= r(q_iB_i-q_oB_o)\end{aligned} \tag{13}$$

i.e. evolution proceeds in the direction of the the strategy that increases expected fitness. However, for intermediate values of θ we can approximate the sigmoidal term as 0 for $l_iB_i+l_oB_o<-n\theta$ and as 1

otherwise. Thus Eq. 12 becomes the sum over the probability distribution conditional on the fact that the payoff received is greater than $-n\theta$. This cannot be calculated explicitly in most cases but note that if $nq_iB_i + nq_oB_o + n\theta > 0$ then terms with low values of l_o or l_i will be eliminated from the summation. Since $q_o < q_i$, this means that players who tend to use out-group interactions will tend to suffer more in this regime, and thus the population becomes risk averse. In contrast, when $nq_iB_i + nq_oB_o + n\theta < 0$ only terms with high values of l_o or l_i will be included in the summation, which tends to favor out-group interactions. This qualitatively captures the results shown in Figure 2.

In the following sections we systematically vary the parameters of Table 2 in order to assess the robustness of the results presented in the main text.

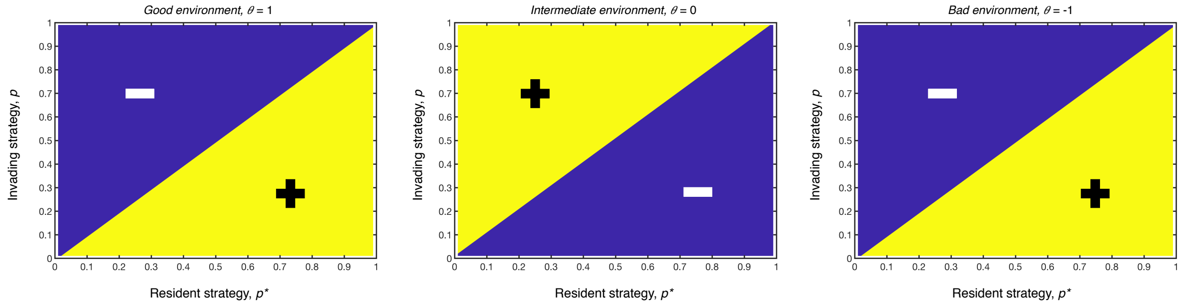

Figure S1 – Pairwise invasability plot in different environments using the default parameters as given in Table 1. We see, just as in Figure 2 of the main text, that low polarization ($p^* = 0$) is stable in a very good or a very bad environment (left and right plots) but that this situation is reversed in an intermediate environment (center plot). Under both local and non-local mutations this effect is evident

2.2 Impact of Risks and Benefits of Interactions

Figure S2 shows the strategies that maximize fitness across environments as we vary the risk parameters q_i and q_o and the benefit parameters B_i and B_o. In all cases we see qualitatively similar results to those shown in Figure 2 of the main text – for intermediate environments risk aversion can lead to an increase in polarization even when the expected benefit of out-group interactions exceed those of in-group interactions.

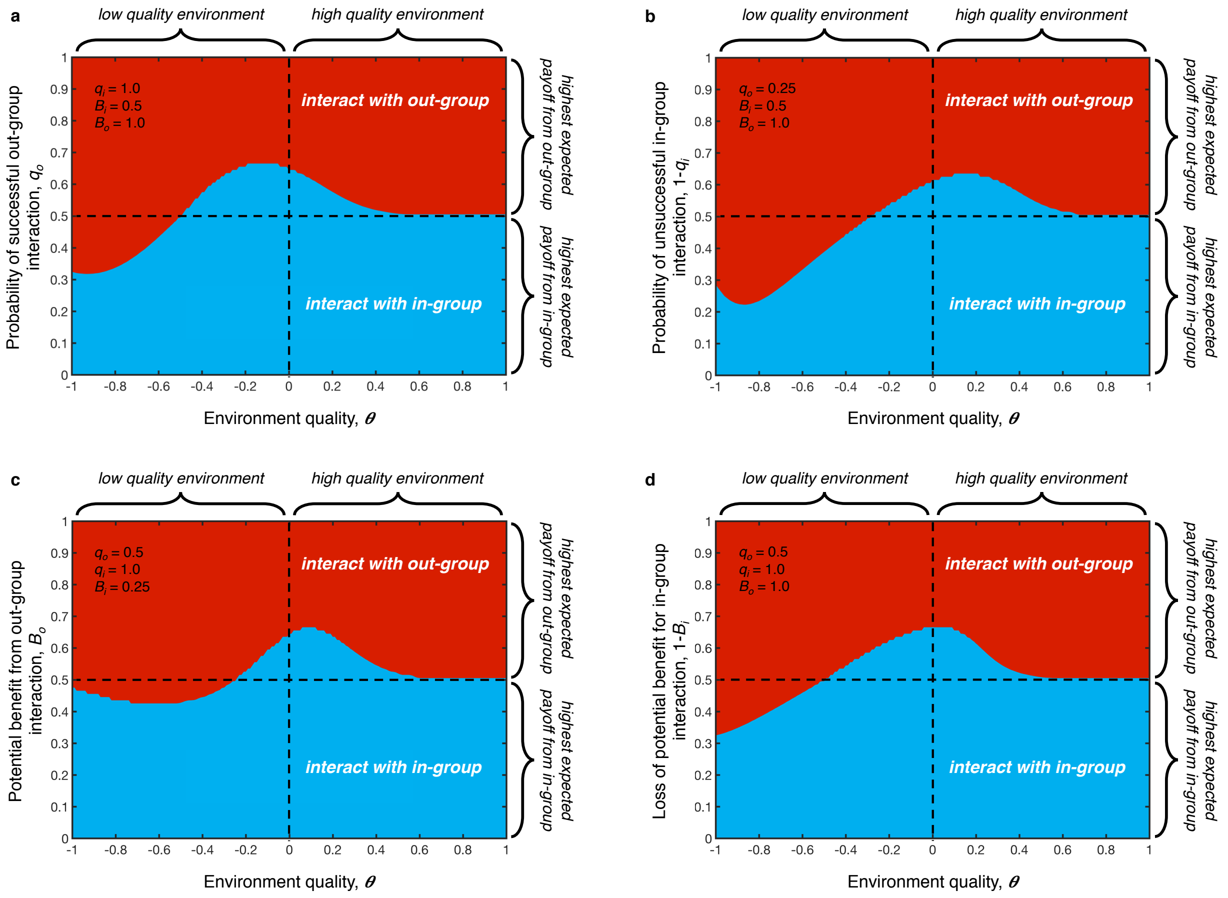

Figure S2 – Stable equilibria as a function of environment (θ, x-axis) and payoff parameters. On the y-axis we vary a) The probability of success of out-group interactions, b) the probability of failure of in-group interactions, c) the benefit of successful in-group interactions and d) the opportunity cost of a successful in-group interaction compared to a successful out-group interaction. All other parameters are set to the default values in Table 1.

We also examined the stable strategies of the model fixing the expected benefits of in- and out-group interactions $B_i q_i$ and $B_o q_o$ and varying the risk associated with out-group interactions. Once again we see a shift from stable low-polarization strategies at intermediate environments, unless the risk of out-group interactions becomes low (i.e q_o becomes sufficiently large) in which case low polarization strategies are always stable.

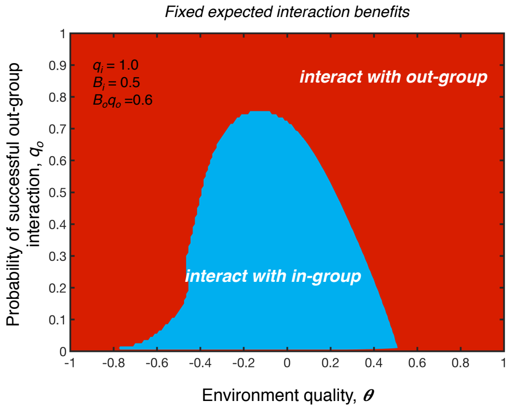

Figure S3 – Stable equilibria assuming fixed expected benefits from in- and out-group interactions, $q_i B_i$ and $q_o B_o$ under varying risk of out-group interactions q_o (y-axis) and across environments (x-axis). All other parameters are set to the default values in Table 1.

2.3 Impact of the Number of Interactions

We examined the effect of interaction number n on our results. From this we draw four qualitative conclusions as follows:

- When the increase in expected benefits per out-group interactions is high (20%) high-polarization can only take hold when the number of interactions is small ($n \in [10, 50]$ interactions, Figure S4a, S4c and S4d))

- When the increase in expected benefits per out-group interactions is low (2%) high-polarization can take hold even when each individual participates in many hundreds of interactions (Figure S4b))

- Increasing the steepness of the sigmoid function (i.e the rate of loss of fitness in a declining environment) makes high polarization more likely to take hold even when individuals participate in many interactions ($n < 50$, Figure S4c)

- Decreasing the steepness of the linear function has qualitatively similar effect (Figure S4d)

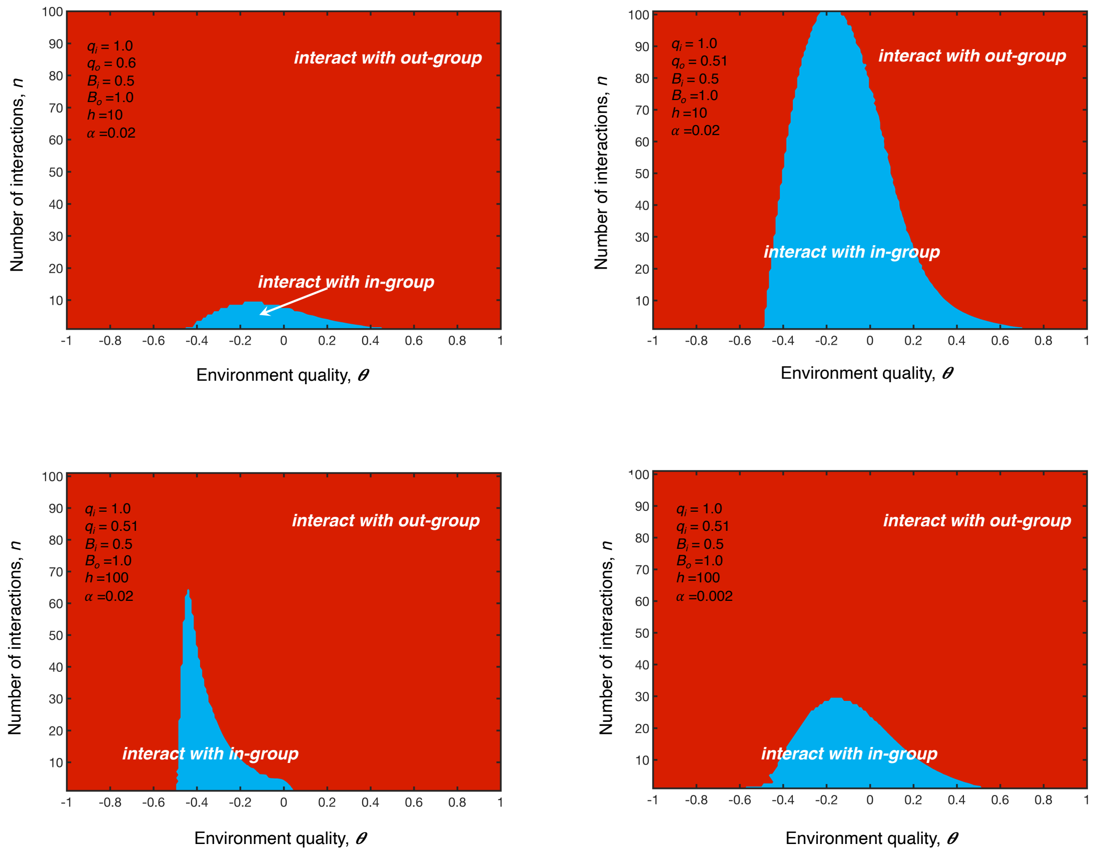

Figure S4 – Stable equilibria with varying numbers of interactions n (y-axis) and across environments (x-axis). (top left) With a 20% increase in expected benefit from out-group interactions compared to in-group interactions high polarization only occurs for $n < 10$ (top right) with a 2% increase however high polarization can take hold even when $n = 100$ (bottom left) With a 20% increase and a steep sigmoidal function ($h = 100$) high polarization can take hold with a greater number of interactions ($n < 50$) and (bottom right) similarly for a shallower linear function $\alpha = 0.002$. All other parameters are as shown in Table 1.

We also consider the case when the number of interactions n is large such that $n \sim N$. In this case we assume that , when using a low-polarization strategy $p = 1$, the number of successful interactions from $n \gg 1$ attempts with the out-group us normally distributed with mean μ and variance σ. We assume that under a low polarization strategy, $p = 0$, all interactions are succesful. Setting $\mu = 0.6$ we explore the stability of in- and out-group strategies as a function of σ (Figure S5).

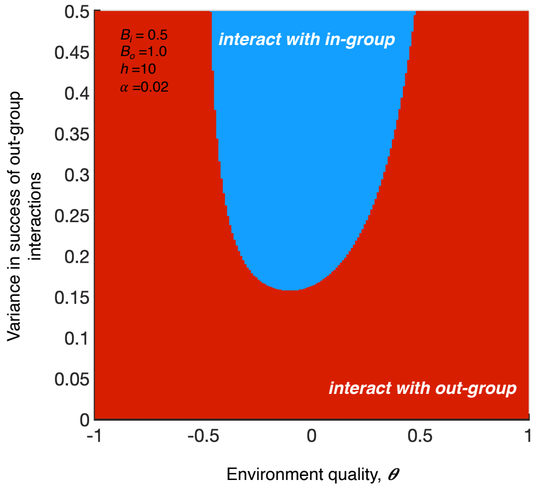

Figure S5 – Stable equilibria with changing variance for a fixed number of interactions $n = 100$ (y-axis) and across environments (x-axis). Once $\sigma > 0.15n$ high polarization can take hold, with the range of environments that favor polarization quickly becoming large once $\sigma > 0.2n$ (blue region). All other parameters are as shown in Table 1.

2.4 Impact of the Rate of Benefit Accumulation

Finally we varied the curvature of the benefit accumulation function. In the main text we assume a function of the form

$$f(l_i, l_o, \theta) = \frac{\exp[h(l_i B_i + l_o B_o + n\theta)/n]}{1 + \exp[h(l_i B_i + l_o B_o + n\theta)/n]}(1 + \alpha(l_i B_i + l_o B_o)) \tag{14}$$

where the first (sigmoidal) term captures the idea that, below a certain threshold fitness rapidly declines either, in a biological context, due to starvation or in an economic context due to in ability to meet basic financial obligations etc. The second (linear) term reflects the fact that, once above the threshold, there is still an advantage to having higher payoff, where h determines the steepness of the threshold function and α the steepness of the linear function. Note that by varying h and α we can produce a whole family of qualitatively different benefit functions from a purely linear function to a Heaviside step function. Finally, note that the position of the threshold above which sufficient benefit from interactions is accumulated depends on the environment θ which describes the harshness of the environment, the cost or availability of resources depending on whether we are thinking about a biological or an human economy.

We see that increasing the steepness of the sigmoid function (Figure S6 - top row) has little effect above $h \sim 10$. However, below this we see an increase in high polarization strategies in good environments. In contrast, increasing the steepness of the linear function α tends to reduce the range of environments in which high polarization strategies can take hold if the expected benefits of out-group interactions exceed those of in-group interactions (Figure S6, middle row).

We also explored the behavior of the model under a benefit accumulation function with constant positive or negative curvature

$$f(l_i, l_o, \theta) = [(l_i B_i + l_o B_o + n\theta)/n]^{10^{\beta}} \tag{15}$$

where we choose the form of the exponent so that $\beta = 0$ corresponds to zero curvature, with negative values corresponding to a concave accumulation function. Here we see as expected that risk-averse, high-polarization strategies only arise when the accumulation function is concave. We also observe the same transition from low- to high- polarization strategies in a declining environment, but without the corresponding reverse transition as the environment continues to decline (since unlike the accumulation function of Eq. 14, Eq. 15 has a fixed direction of curvature – Figure S6, bottom row).

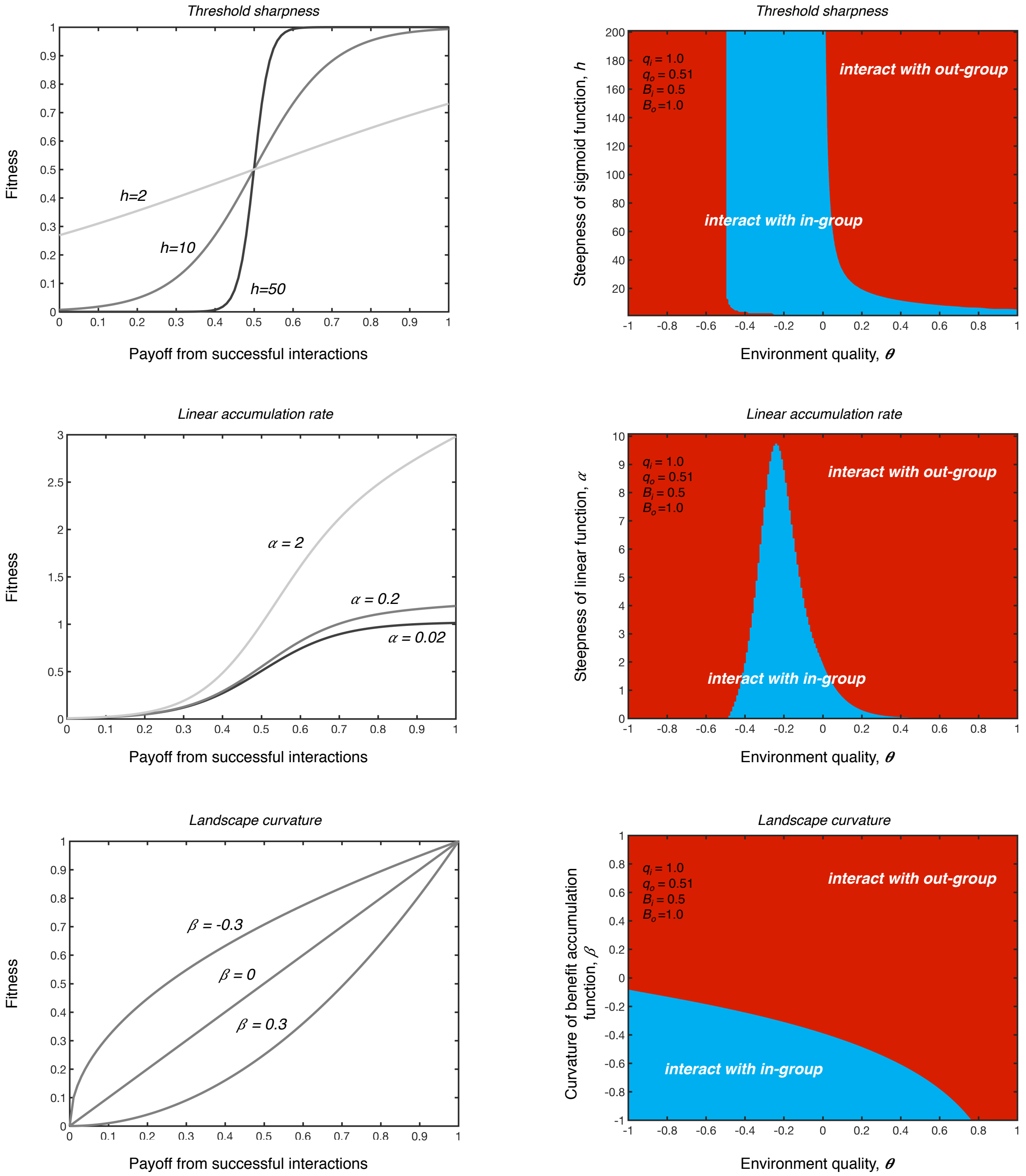

Figure S6 – Stable equilibria for the model with varying benefit accumulation functions. The left hand column shows how the varied parameter changes the shape of the accumulation function, while the right hand column shows the equilibria for the model as the parameter varies (y-axis) across different environments (y-axis). Top row – Increasing the steepness of the sigmoidal function has little impact above $h = 10$ however for smaller values we see an increase in polarization in good environments. Middle row – Steeper linear components to the accumulation function Eq. 14 tend to decrease the range of environments when polarization can take hold. Bottom row – Varying the curvature of the accumulation function Eq.15 demonstrates the known result that risk aversion requires a concave utility function, where we see the same transition from low to high polarization as environments decline as described in the main text. parameter values are as shown in Table 1, with the exception that we have set $q_o = 0.51$ to make the impact of varying these parameters more clearly visible.

3 Case 2: Social payoffs

3.1 Stability and Invasibility

Under this model the probability of success of an out-group interaction for a player h interacting with another player g is $q_o(1-p_g)$ where the term $(1-p_g)$ accounts for the willingness of g to engage in an out-group interaction. We assume that players are always willing to engage in an in-group interaction if initiated by another player (which captures the idea that players are always willing to share ideas etc with members of their group. This may not be the case if such interactions are intrinsically costly). Pairwise invasibility plots for the model are shown in Figure S7-8 below.

Because we are assuming a population in which $N \gg n$, the fitness of the resident g is independent of the mutant f, such that the selection gradient only depends on w_f, which gives us Eq. 6 in the main text. Calculating this gradient explicitly by differentiating Eq. 6 of the main text gives

$$\frac{\partial s(f,g)}{\partial p_f} = \sum_{k=0}^{n}\sum_{l_i=0}^{k}\sum_{l_o=0}^{n-k}\binom{n}{k}p_f^{k-1}(1-p_f)^{n-k-1}(k-np_f)\times \binom{k}{l_i}q_i^{l_i}(1-q_i)^{k-l_i}\binom{n-k}{l_o}(q_o(1-p_g))^{l_o}(1-q_o(1-p_g))^{n-k-l_o}\times \frac{\exp[h(l_iB_i+l_oB_o+n\theta)]}{1+\exp[h(l_iB_i+l_oB_o+n\theta)]}(1+r(l_iB_i+l_oB_o)) \tag{16}$$

which once again we can evaluate numerically to calculate the points of zero selection gradient as shown in Figure 3 of the main text and below. However we can also evaluate Eq. 16 in the special case where the environment is sufficiently bad that $l_iB_i + l_oB_o + n\theta < 0, \ \ \forall\ l_i, l_o$ or sufficiently good $l_iB_i + l_oB_o + n\theta > 0, \ \ \forall\ l_i, l_o$ so that we can approximate the sigmoidal term in Eq. 17 as constant and recover selection gradient

$$\begin{aligned}\frac{\partial s(f,g)}{\partial p_f} &= \sum_{k=0}^{n}\binom{n}{k}p_f^{k-1}(1-p_f)^{n-k-1}(k-np_f)(1+nrq_oB_o+kr(q_iB_i-q_oB_o))\\ &= r(q_iB_i - q_o(1-p_f)B_o)\end{aligned} \tag{17}$$

which, when evaluated at $p_f = p_g$ means that the invasion success of the mutant depends on the resident strategy. In particular there is an equilibrium at $p^* = 1-\frac{q_iB_i}{q_oB_o}$, which is always a viable strategy provided $q_oB_o > q_iB_i$ i.e out-group interactions have higher intrinsic expected payoff than in-group interactions.

We can evaluate the stability of this equilibrium by taking the second derivative (see Eq. 11 above) which gives

$$\frac{\partial^2 s(f,g)}{\partial f^2} = rq_oB_o. \tag{18}$$

We see that the equilibrium is always unstable. In addition we note that at the upper boundary, when $p_f = p_g = 1$ Eq. 17 reduces to rq_iB_i which is always positive, indicating maximum polarization is always stable in extreme environments under this model. Similarly, at the lower boundary when $p_f = p_g = 0$ Eq. 17 reduces to $q_iB_i - q_oB_o$ which is always negative if the expected intrinsic payoff from out-group interactions is greater than from in-group interactions.

Finally in the case of intermediate environments Eq. 17 cannot be analyzed explicitly, although it can be explored numerically as shown in Figure 3 of the main text and in Figure S7 below.

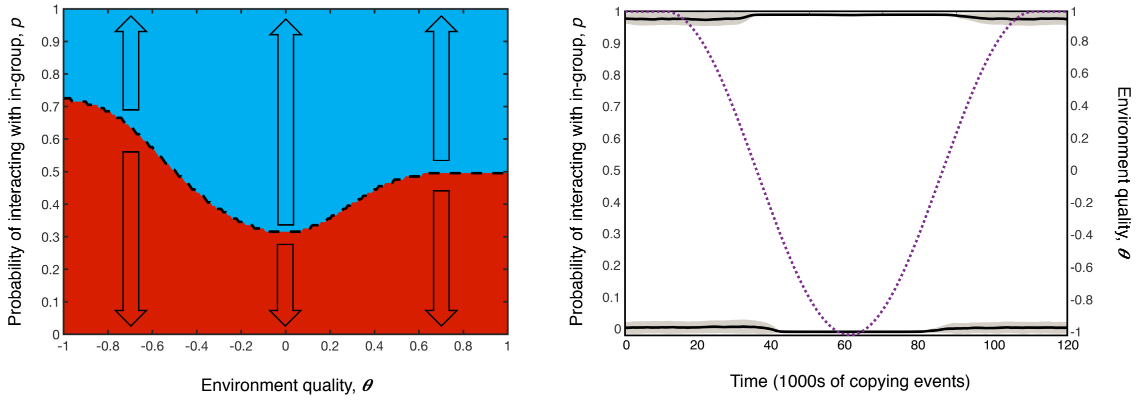

Figure S7– Model of polarization under which the success of an out-group interaction depends on *both* the intrinsic probability of success, q_o and the willingness of other players to engage in out-group interactions, $1 - p$ i.e. on the strategy of other members of the group or population, as shown in Figure 3 of the main text, with in-group interaction benefit $B_1 = 0.2$. (left) Under the framework of adaptive dynamics, we calculate the selection gradient for invasion by a rare, local mutant in a monomorphic population, We calculate the selection gradient as a function of environment quality θ and of resident population strategy p. The direction of the selection gradient and the consequent evolutionary dynamics are shown by the blue (increasing polarization) and red (decreasing polarization) regions with arrows indicating the direction of evolutionary change in p for a given environment θ (right). We show that under these parameters, the system is *bistable* across all environments b) As a result, a population initialized at a low or high polarization state tends to remain there (black line) regardless of the environment (purple dashed line) tends Each individual is assigned to one of two groups so that all individuals have an in- and an out-group of 500 individuals under the assumptions of the adaptive dynamics model described above (see main text). Innovations, in which individuals try out novel strategies, occur at rate $\mu = 0.001$ per copying event and new strategies occurred via a deviation around the current strategy of size $\Delta = 0.01$, plus boundary conditions to ensure strategies remain in the physical range $[0, 1]$. Model parameters and visualization are otherwise as per Figure 2b.

However the stability at the boundaries can be assessed. Taking $p_g = 1$ we recover

$$\frac{\partial s(f,g)}{\partial p_f} = nq_i^n \frac{\exp[h(nB_i + n\theta)]}{1+\exp[h(nB_i + n\theta)]}(1 + rnB_i) +$$
$$\sum_{l_i=0}^{n-1} \binom{n-1}{l_i} q_i^{l_i}(1-q_i)^{n-1-l_i} \left(\frac{n^2(1-q_i)}{n-l_i} - 1\right) \frac{\exp[h(l_iB_i + n\theta)]}{1+\exp[h(l_iB_i + n\theta)]}(1 + rl_iB_i)$$

Now note that if this quantity is positive when the sigmoidal term is constant it is always positive as the sigmoidal term will always reduce the contribution of terms $l_i < n(1 - n(1 - q_i))$ that contribute negative weight to the summation more than it reduces terms $l_i > n(1 - n(1 - q_i))$ thus we can assess the stability of the upper boundary by settling the sigmoid constant and equal to 1. We then find from Eq. 18

$$\frac{\partial s(f,g)}{\partial p_f} = rq_iB_i$$

as given above. Thus the upper boundary is always stable except in the limit $q_iB_i \to 0$ in which case the upper boundary converges with the unstable point p^*.

Finally we consider the stability of the lower boundary $p_g = 0$ at which point we find selection gradient

$$\begin{aligned}\frac{\partial s(f,g)}{\partial p_f} = &\sum_{l_i=0}^{1}\sum_{l_o=0}^{n-1} q_i^{l_i}(1-q_i)^{1-l_i}\binom{n-1}{l_o} q_o^{l_o}(1-q_o)^{n-1-l_o} \times \\ &\frac{\exp[h(l_iB_i + l_oB_o + n\theta)]}{1+\exp[h(l_iB_i + l_oB_o + n\theta)]}(1 + r(l_iB_i + l_oB_o)) - \\ &\sum_{l_o=0}^{n} n\binom{n}{l_o} q_o^{l_o}(1-q_o)^{n-l_o} \frac{\exp[h(l_oB_o + n\theta)]}{1+\exp[h(l_oB_o + n\theta)]}(1 + r(l_oB_o))\end{aligned} \tag{19}$$

which can be both positive and negative as illustrated in Figure 3 of the main text and below. Finally we also note that the form of Eq. 17 permits the existence of equilibria for non-boundary values of p_g, such that the system contains multiple stable equilibria. We given an example of such a case below.

3.2 Bi-stability and Multi-stability

The qualitative difference between the first (Case 1) and second (Case 2) models presented in Figure 2 of the main text is the ability of the Case 2 model to sustain multiple stable equilibria. This is shown for the case of local mutations under the framework of adaptive dynamics. However pairwise invasion plots for the same parameter values used to produce Figure 3 of the main text reveal that both equilibria are in fact stable against all invaders (Figure S8). This leads to our conclusion that Case 2 can produce irreversible loss of low-polarization behavior absent coordinated behavioral shifts that bypass the disadvantage faced by rare invaders.

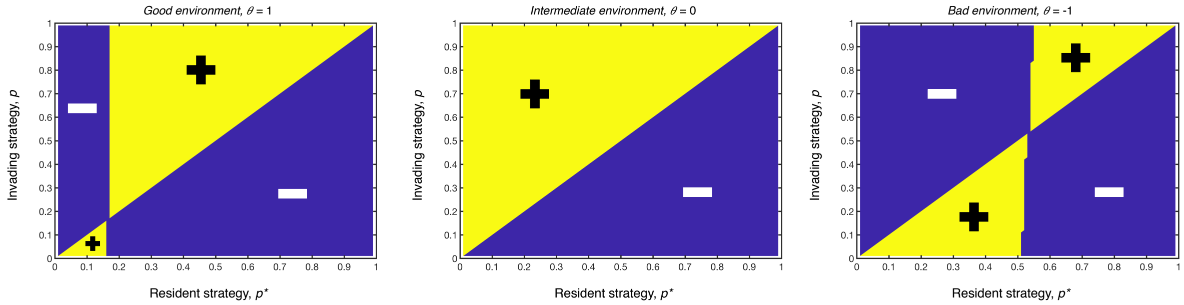

Figure S8 – Pairwise invasability plot in different environments using the default parameters as given in Table 1. We see, just as in Figure 3 of the main text, that low polarization ($p^* = 0$) and high polarization ($p^* = 1$) are both stable in a very good or a very bad environment (left and right plots) but that only high polarization is stable for this choice of parameters in an intermediate environment, which can lead to the irreversible loss of low polarization behavior in a shifting environment. As shown in above, the high polarization equilibrium is never lost, although its basin of attraction can become arbitrarily small

We also note that Eq. 17 permits the possibility of equilibria that lay in the interior of strategy space i.e for values $0 < p_g < 1$ of the resident strategy. We illustrate the existence of such a stable interior equilibrium in Figure S9 below, and also note its vulnerability to environmental shifts which disrupt the equilibrium and lead to invasion by high or low polarization strategies.

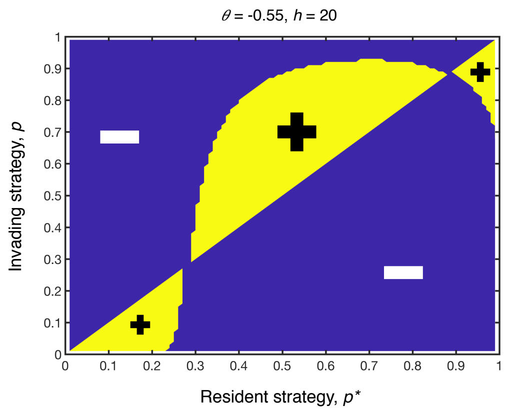

Figure S9– Pairwise invasability plot showing three stable equilibria. Two are globally stable, at $p^* = 0$ (low polarization()and $p^* \approx 0.88$ (high intermediate polarization) while the high polarization equilibrium is seen to be locally stable. The parameters shown are as given in Table 1, with the alteration that a steep threshold ($h = 100$) is required to generate the internal equilibrium.

3.3 Rising inequality and economic decline

To supplement main text Figure 3 we ran simulations in which a smaller proportion of the population, $\pi = 0.01$ and $\pi = 0.001$, are wealthy. We also ran simulations assuming that the average environment θ_0 was increasing while the level of wealth inequality θ_g was decreasing (and vice versa). As shown in Figure S10 below, both cases lead to the emergence and entrenchment of polarization as in Figure 3.

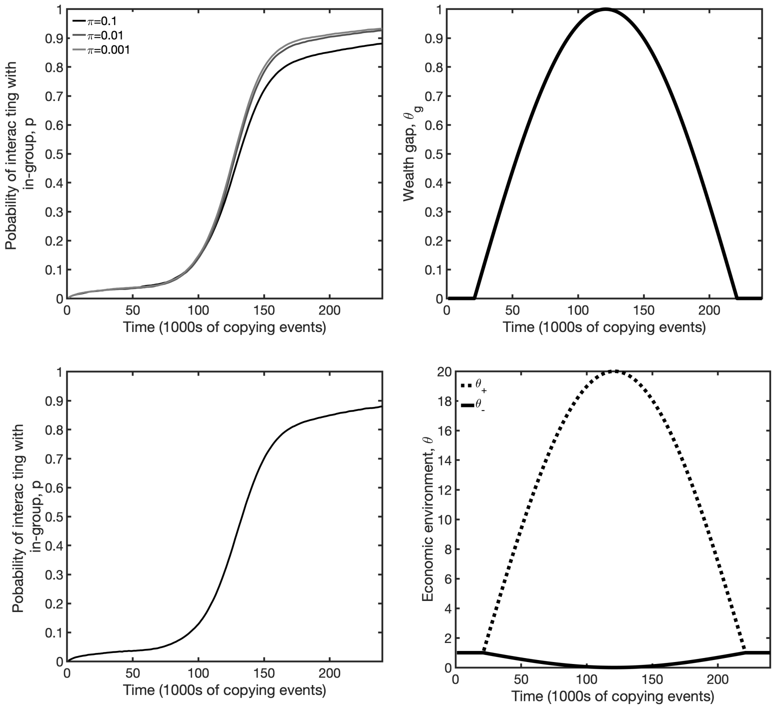

Figure S10 – (top row) We ran individual-based simulations in an economic environment with exogenously changing levels of inequality θ_g with θ_0 fixed and varying π. Here we assume sinusoidally varying inequality with $\theta_0 = 1.0$ and $\theta_g \in [0, 1.0]$ and different values of π as shown in the legend. All other parameters are those provided in main text Figure2 c-d (top left) in all three cases the population behavior is qualitatively the same, with polarization emerging and becoming entrenched as (top right) inequality θ_g in creases. (bottom row) We ran individual-based simulations in an economic environment with exogenously changing levels of inequality θ_g and θ_0 with fixed $\pi = 0.1$. Here we assume sinusoidally varying environment such that $\theta_0, \theta_g \in [0, 1.0]$ and with $theta_g$ increasing as θ_0 decreases. All other parameters are those provided in main text Figure 2c-d (bottom left) The population behavior is qualitatively the same as in the other cases, with polarization emerging and becoming entrenched as (bottom right) the environment of the wealthy θ_+ increases (dashed line) wile the environment of the non-wealthy θ_- decreases (solid line) and vice versa.

4 Individual-based Simulations

We performed individual based simulations to test the analytical and numerical predictions from the model presented in the main text and in this supplement. Simulations were performed under the copying process using populations of $N = 1000$ individuals with the mean trajectories determined from an ensemble of 10^4 sample paths. Simulations were run for $100N - 200N$ copying events and environmental shifts were simulated by allowing θ to change sinusoidally with a period of $100N - 200N$ copying events. Fitness was calculated for each individual by randomly assigning all members of the population to one of two groups. To simulate Case 2 out-group interactions were then determined for a given focal individual by randomly choosing a player not from their group with the success of the interaction determined by the chosen player's strategy and the intrinsic success rate q_o. Mutations were assumed to occur at a rate $0.1N$ per copying event, with the target of the mutation chosen randomly from the population. For the non-social model (Case 1) simulations, we allowed global mutations such that the mutating player was assigned a new strategy $p^\dagger \in [0, 1]$. For the Case 2 simulations, we used local mutations such that the target of the mutation had their strategy perturbed by $\Delta = \pm 0.01$ with mutations that increase and decrease p equally likely, and we impose the appropriate boundary conditions to ensure strategies were physical.

5 Data analysis

Here we provide additional analysis and robustness checks for the empirical results presented in the main text (Figure 4). We consider the relationship between affective polarization and inequality at the state level in the USA, across the last three presidential election cycles, 2008-2016. We use data from the ANES and CCES election surveys to measure affective polarization (**????**) along with data from the Census Bureau and the Federal Elections Commission on Gini coefficients, unemployment rates and vote share at the time of each election in each state (**?**).

Affective polarization is defined at the individual level as the difference in "warmth", measured via a "feelings thermometer", between the major party an individual most identifies with (Republican or Democrat) and the out-party, i.e. the major party the respondent least identifies with. Feelings thermometers have long been a standard part of election surveys, and are administered on a 100 point scale, with 0 corresponding to strong negative feelings towards a party, and 100 corresponding to strong positive feelings. Intuitively, if an individual gives a high score to one party and a low score to another, this indicates a high degree of affective polarization i.e. a large net positive feeling towards a preferred party.

Recent research in political science has stressed that partisanship is a salient social identity as well

as a marker for the various other group identities that have become associated with the major parties (**????**). Thus, affective polarization is a measure well-suited to evaluating our group-based model.

Individual-level affective polarization scores can be used to calculate an average at the state or national level, which provides a measure of the degree to which the electorate is polarized in their attitudes towards the parties. The in- or out-group favoring behavioral strategies described by our model can be understood as corresponding to this individual-level affective polarization. Since we predict (Figure 3) that inequality can act as a driver of in-group favoring attitudes, we can seek support for this prediction in the affective polarization data. To this end we present three analyses which support the hypothesis that inequality can act as a driver for affective polarization.

5.1 Pooled data

In Figure 4 (main text) we show the correlation between the average polarization at the state level and the state-level Gini coefficient. Results shown are for the pooled data across all three election cycles (50 states + DC for each of three election cycles, for a total of 153 data points). This correlation is significant under a two-way fixed effects model with a single intercept (two-tailed t-test, $t = 5.2$, $p < 0.01$). The state-level data for each election cycle is displayed in Table S2 below, and the code to reproduce the analysis is available via github.

State	2008 sample size	2012 sample size	2016 sample size	2008 affective polarization	2012 affective polarization	2016 affective polarization	2008 Gini coefficient	2012 Gini coefficient	2016 Gini coefficient	2008 unemployment rate	2012 unemployment rate	2016 unemployment rate	2008 Republican vote share	2012 Republican vote share	2016 Republican vote share
AL	18	77	62	0.37	0.54	0.51	0.467	0.4726	0.4847	5.9	8.1	5.8	0.61	0.61	0.64
AK	0	3	7	-	0.25	0.28	0.403	0.4232	0.4081	6.7	7	6.9	0.61	0.57	0.58
AZ	39	99	159	0.22	0.4	0.4	0.453	0.4608	0.4713	6.6	8.2	5.3	0.54	0.55	0.52
AR	0	32	57	-	0.29	0.46	0.459	0.4632	0.4719	5.5	7.6	3.9	0.6	0.62	0.64
CA	258	646	601	0.35	0.4	0.44	0.473	0.4822	0.4899	7.6	10.2	5.4	0.38	0.38	0.34
CO	84	75	128	0.36	0.36	0.4	0.457	0.4579	0.4586	5	7.8	3.2	0.45	0.47	0.47
CT	26	48	90	0.31	0.42	0.39	0.486	0.4915	0.4945	5.9	8.5	5.1	0.39	0.41	0.43
DE	15	22	20	0.44	0.42	0.43	0.447	0.4356	0.4522	5.2	7.3	4.5	0.37	0.41	0.44
DC	11	9	35	0.53	0.71	0.47	0.54	0.5343	0.542	6.5	8.8	6	0.07	0.07	0.04
FL	144	365	382	0.32	0.44	0.44	0.471	0.4828	0.4852	6.7	8.4	4.8	0.49	0.5	0.51
GA	63	150	183	0.37	0.39	0.47	0.467	0.4809	0.4813	6.3	9	5.3	0.53	0.54	0.53
HI	0	4	16	-	0.3	0.24	0.428	0.4257	0.442	4.6	5.8	2.9	0.27	0.28	0.33
ID	0	12	60	-	0.36	0.32	0.419	0.4302	0.4503	5.8	7.1	3.8	0.63	0.66	0.68
IL	27	203	259	0.45	0.44	0.35	0.467	0.4716	0.481	6.5	9	5.7	0.37	0.41	0.41
IN	43	90	128	0.44	0.39	0.39	0.437	0.4417	0.4527	6.1	8.3	4.3	0.49	0.55	0.6
IA	0	59	66	-	0.38	0.4	0.428	0.4328	0.4451	4.3	5	3.6	0.45	0.47	0.55
KS	23	26	91	0.28	0.38	0.35	0.442	0.4496	0.455	4.7	5.6	4.1	0.58	0.61	0.61
KY	0	68	79	-	0.42	0.47	0.467	0.4667	0.4813	6.7	8.2	5.1	0.58	0.62	0.66
LA	73	97	79	0.36	0.53	0.37	0.477	0.4859	0.499	5.2	6.8	6.1	0.59	0.59	0.6
ME	0	8	27	-	0.31	0.4	0.436	0.445	0.4519	5.6	7.4	3.8	0.41	0.42	0.48
MD	0	86	131	-	0.41	0.42	0.438	0.4473	0.4499	4.5	7	4.4	0.37	0.37	0.36
MA	21	109	178	0.35	0.35	0.45	0.471	0.4813	0.4786	5.8	6.6	3.7	0.37	0.38	0.35
MI	97	143	203	0.48	0.43	0.44	0.451	0.4624	0.4695	8.2	9.1	5	0.42	0.45	0.5
MN	22	109	113	0.35	0.42	0.39	0.445	0.4441	0.4496	5.6	5.6	4	0.45	0.46	0.49
MS	55	30	63	0.45	0.46	0.43	0.478	0.487	0.4828	6.9	9	5.8	0.57	0.56	0.59
MO	0	92	97	-	0.42	0.42	0.448	0.4608	0.4646	6.4	6.8	4.8	0.5	0.55	0.6
MT	0	22	15	-	0.4	0.49	0.449	0.4502	0.4667	5.3	5.9	4.1	0.51	0.57	0.61
NE	0	31	32	-	0.32	0.37	0.427	0.4339	0.4477	3.4	4	3.1	0.58	0.61	0.64
NV	27	48	53	0.34	0.49	0.42	0.431	0.4516	0.4577	7	11	5.6	0.44	0.47	0.49
NH	0	11	50	-	0.39	0.43	0.419	0.4298	0.4304	3.8	5.6	2.9	0.45	0.47	0.5
NJ	20	124	166	0.36	0.4	0.44	0.462	0.4718	0.4813	5.4	9.4	5	0.42	0.41	0.43
NM	52	53	51	0.36	0.39	0.41	0.46	0.4713	0.4769	4.5	7	6.7	0.42	0.45	0.45
NY	99	268	303	0.35	0.4	0.4	0.503	0.5009	0.5129	5.5	8.5	4.9	0.36	0.36	0.38
NC	60	196	220	0.39	0.45	0.44	0.463	0.4691	0.478	6.1	9.2	5	0.5	0.51	0.52
ND	34	8	14	0.27	0.5	0.52	0.451	0.4597	0.4533	3.2	3.1	3	0.54	0.6	0.7
OH	75	194	244	0.35	0.4	0.41	0.45	0.4615	0.468	6.7	7.2	5	0.48	0.48	0.54
OK	34	54	86	0.4	0.45	0.35	0.458	0.4643	0.4645	3.8	5.2	4.9	0.66	0.67	0.69
OR	25	71	84	0.4	0.35	0.46	0.447	0.4565	0.4583	6.7	8.7	4.8	0.42	0.44	0.44
PA	32	236	292	0.31	0.42	0.39	0.457	0.4642	0.4689	5.4	7.9	5.5	0.45	0.47	0.5
RI	14	17	17	0.35	0.31	0.45	0.46	0.4647	0.4781	8.2	10.2	5.2	0.36	0.36	0.42
SC	49	106	96	0.4	0.44	0.43	0.464	0.4684	0.4735	7.1	9	4.9	0.55	0.55	0.57
SD	0	10	12	-	0.19	0.31	0.446	0.4335	0.4495	3.2	4.3	3	0.54	0.59	0.66
TN	66	87	173	0.37	0.38	0.38	0.471	0.4732	0.479	6.9	7.8	4.8	0.58	0.6	0.64
TX	303	459	429	0.33	0.42	0.4	0.474	0.4767	0.48	4.9	6.6	4.7	0.56	0.58	0.55
UT	0	42	27	-	0.33	0.34	0.411	0.4244	0.4263	3.6	5.2	3.4	0.65	0.75	0.62
VT	0	6	11	-	0.47	0.33	0.434	0.4392	0.4539	4.7	5	3.2	0.31	0.32	0.35
VA	28	106	133	0.3	0.43	0.39	0.46	0.4661	0.4705	4	6	4.2	0.47	0.48	0.47
WA	22	128	133	0.32	0.33	0.41	0.442	0.4498	0.4591	5.5	8	5.2	0.41	0.42	0.41
WV	0	29	35	-	0.3	0.36	0.453	0.4638	0.4711	4.1	7.7	6	0.57	0.64	0.72
WI	20	109	139	0.37	0.38	0.43	0.426	0.4401	0.4498	4.9	7	4	0.43	0.46	0.5
WY	0	2	9	-	0.12	0.39	0.441	0.4166	0.436	3.1	5.4	5.3	0.67	0.71	0.76

Table S2 – Pooled data for each state during each presidential election cycle 2008-2016. Affective polarization is calculated as the average across the respondents within the state during the election cycle and normalized to produce a value in the range $[0, 1]$.

5.2 Election-specific intercepts

Next we consider the pooled data under a two-way fixed effects model with election-specific intercepts, which accounts for the systematic change in Gini coefficient across states over the 8-year period 2008-2016. This correlation is significant when all data is included (two-tailed t-test, $t = 5.7$, $p < 0.01$). When we exclude states with fewer than 10 respondents in a given election cycle the correlation remains significant (two-tailed t-test, $t = 4.4$, $p < 0.01$ – this is the case shown in Figure S11) and the result continues to hold when states with fewer than 50 respondents are excluded (two-tailed t-test, $t = 2.1$, $p < 0.05$).

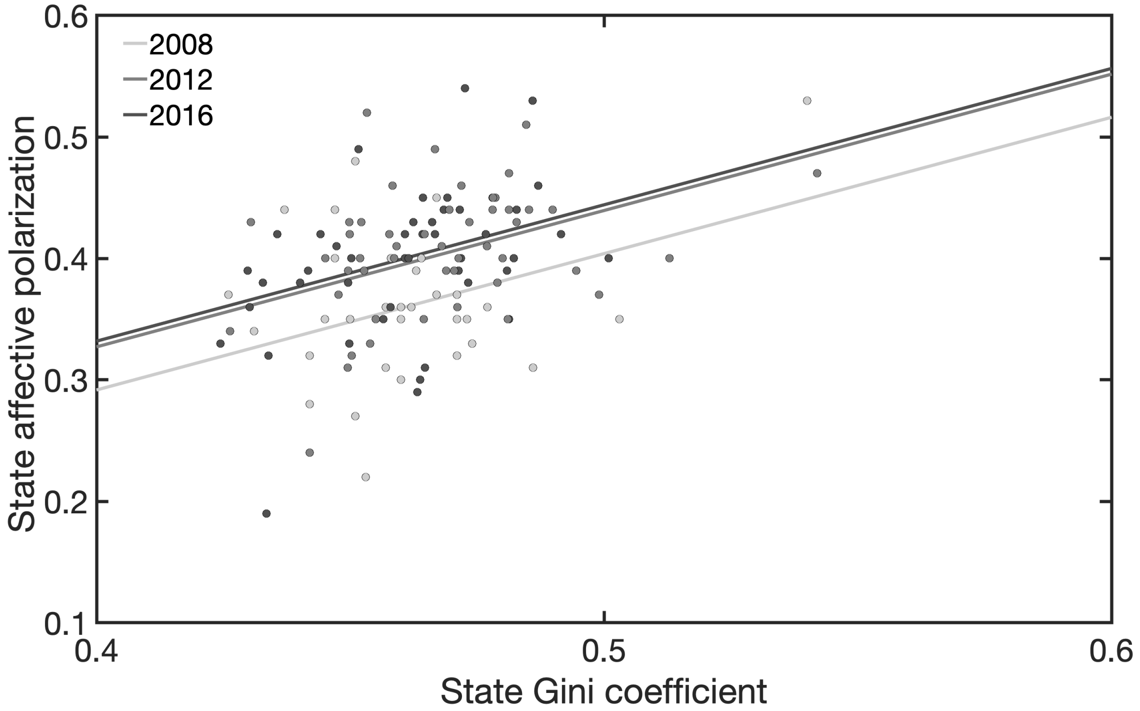

Figure S11 – We show the correlation for the pooled data across all three election cycles between state-level affective polarization estimated from (????) and state-level Gini coefficient taken from (?), under a fixed-effects model with election-specific intercepts. Data shown exclude states with fewer than 10 respondents.

5.3 Individual-level data

Finally we look at the correlation between Gini and affective polarization at the level of the individual respondent, controlling for the effect of race and education of the individual and unemployment rate, and partisan lean (measured by the percentage vote received by Republicans vs Democrats in the corresponding presidential election) of the state where they reside. Race is encoded as a dummy variable (white vs non-white) while education is measured on a 6-point scale under the standard election survey. Gini, unemployment rate and partisanship for each individual are given according to the value

for the state at the time of the election. The results are shown in Table S3 with Gini and education showing a significant positive individual-level correlation with affective polarization, and race showing a significant negative correlation. Race and education both display strong correlations, but are also individual-specific measures. This indicates that the background level of inequality an individual experiences is positively correlated with the degree of polarization they express, but this effect is strongly modulated by individual-level variations in race and education.

**Independent variables**	**Correlation**	**p-value**
GINI	0.58* (0.16)	0.0003
Unemployment rate	0.0002 (0.001)	0.87
Vote share (R/D)	0.05 (0.03)	0.11
Race (white/non-white)	-0.04* (0.005)	7×10^{-16}
Education	0.009* (0.003)	0.0002
N	13045	-
Adjusted R^2	0.00706	-

Table S3 – Individual-level correlations. The second column shows the correlation coefficient along with the standard error in parentheses. Correlations significant with $p < 0.01$ are indicated with an asterisk, while the calculated p-values are shown in the third column.
